# Migration of BTEX and Biodegradation in Shallow Underground Water through Fuel Leak Simulation

**DOI:** 10.1155/2016/7040872

**Published:** 2016-11-01

**Authors:** Yaping Cheng, Yudao Chen, Yaping Jiang, Lingzhi Jiang, Liqun Sun, Liuyue Li, Junyu Huang

**Affiliations:** ^1^College of Environmental Science and Engineering, Guilin University of Technology, Guilin 541004, China; ^2^Guangxi Scientific Experiment Center of Mining, Metallurgy and Environment, Guilin University of Technology, Guilin 541004, China

## Abstract

To provide more reasonable references for remedying underground water, fuel leak was simulated by establishing an experimental model of a porous-aquifer sand tank with the same size as that of the actual tank and by monitoring the underground water. In the tank, traditional gasoline and ethyl alcohol gasoline were poured. This study was conducted to achieve better understanding of the migration and distribution of benzene, toluene, ethyl benzene, and xylene (BTEX), which are major pollutants in the underground water. Experimental results showed that, compared with conventional gasoline, the content peak of BTEX in the mixture of ethyl alcohol gasoline appeared later; BTEX migrated along the water flow direction horizontally and presented different pollution halos; BTEX also exhibited the highest content level at 45 cm depth; however, its content declined at the 30 and 15 cm depths vertically because of the vertical dispersion effect; the rise of underground water level increased the BTEX content, and the attenuation of BTEX content in underground water was related to the biodegradation in the sand tank, which mainly included biodegradation with oxygen, nitrate, and sulfate.

## 1. Introduction

With the development of the oil industry, petroleum leakage during petroleum use, transport, and storage has resulted in serious water pollution from fuel hydrocarbon, which became a substantial threat to underground water safety [1]. At the beginning of 1990s, 90,000 out of 2 million underground gasoline tanks were found with leaks in the United States [2, 3]. According to statistics, 70% of underground gasoline tanks with service lives of over 15 years are more likely to leak [4] and filling stations are potential pollution source of organic pollution. This concern has then become a global problem [5, 6]. Benzene, toluene, ethyl benzene, and xylene (BTEX) are prime contaminants that have attracted wide attention because of their high water solubility and toxicity. BTEX can cause cancers, mucosal pain, blood diseases, damage to the central nervous and respiratory systems, and liver and kidney functional impairment [7, 8]. Six benzene series including BTEX were placed on the top blacklist of pollutants for priority control in China [9]. In China, underground water pollution derived from petroleum use and other activities is also fairly common. For example, BTEX was detected in the shallow groundwater of Yangtze River Delta [10] and benzene series was discovered in Taihu Lake Basin [11], Nanyang Oilfield, Liulin Spring in Shanxi [12], and the Shuanghe River and Weigang water sources near Henan Oilfield [13].

A porous-aquifer sand tank with similar size as that of the actual tank was constructed in our laboratory for simulation experiments on fuel leakage after traditional petroleum and ethanol gasoline pouring. The underground water was monitored for a long period to determine the migration features of BTEX in underground water and to provide the literature for pollution control and water recovery.

## 2. Materials and Methods 

### 2.1. Sand Tank Model

**Figure 1:**
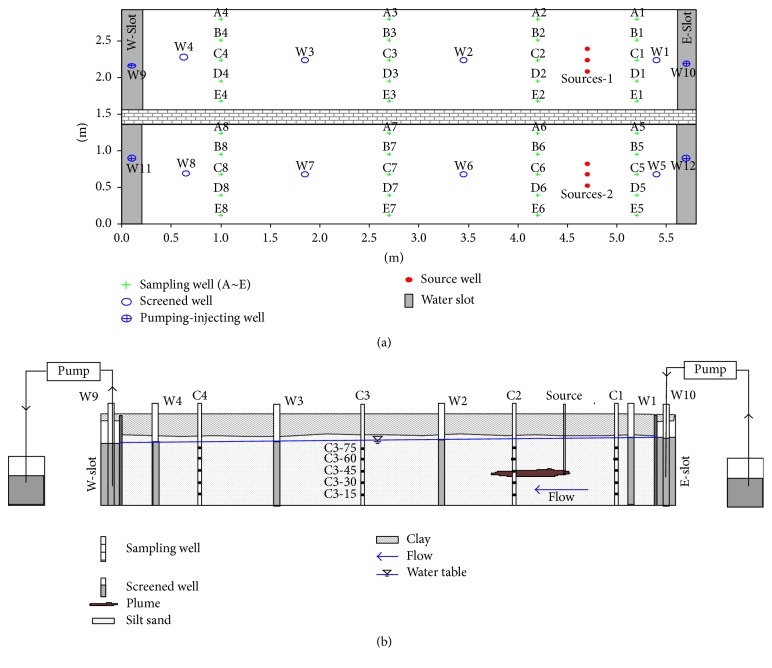
Experimental plan view and cross-sectional view of the sand tank.

The constructed sand tank (Figure 1) was 5.8 m long in a brick-concrete structure with antiseepage. The tank included two narrow-slit water sinks of 0.25 m length on two ends and a 5.3 m long porous aquifer. The tank was 2.9 m wide. For comparative analysis, the tank was divided into two parts by a 0.2 m thick wall, and the porous aquifers on the north and south ends were 1.36 m in width. The diameter of the pure fine sands in this tank ranged from 0.25 mm to 0.05 mm. Oil orifices, specifically multilayered (five layers of 15, 30, 45, 60, and 75 cm heights, resp.) sampling observation holes and a water-level observation hole, were designed.

### 2.2. Experimental Setup

This experiment was performed on July 13, 2015. The water level was 50 cm when the fuel was poured through orifices sw-1 and sw-2. A hose was placed in the tank and the hose's bottom altitude was 45 cm, which corresponded to the level of the 45 cm layered sample tap. Before the experiment was commenced, the average background concentrations of the constituents in the tank were 4.2 mg/L nitrate, 14.3 mg/L sulfate, 4.0 mg/L chloridion, 1.5 mg/L acetate, and 4.1 mg/L dissolved oxygen, at pH 7.6 and 25.9°C. To stimulate the underground water flow, water was directed to flow in from the east side and flow out from the west side at an initial constant flow of 30 mL/min and an average hydraulic slope of around 0.0013. Furthermore, the pumping-filling cyclic experiment was performed, when the porosity was 0.3 at a speed of 30 mL/min and Darcy's underground water velocity of 0.07 m/d (actual flow is 0.24 m/d). After MODFLOW simulation, the seepage was observed as a one-dimensional horizontal flow. To determine the migration and distribution features of gasoline and ethanol gasoline in underground water, we poured 3 L traditional gasoline through sw-1 (northern part) and 3 L 10% ethanol gasoline in sw-2 (southern part) at the speed of transfusion (about 500 mL/h). Meanwhile, 1 L of the 500 mg/L KBr solution was filled in two parts as tracer agent. To maintain their synchronization, the speed of the poured KBr solution was 1/3 that of the poured gasoline.

### 2.3. Sampling and Analysis

Fifteen milliliters of water was sampled from each sample hole with injectors, and 0.5 mL mercuric chloride (800 mg/L) was used for sterilization. Five milliliters of water was placed in a headspace bottle sealed by an aluminium cap for ethyl alcohol and BTEX determination. Meanwhile, 10 mL water was placed in a Dionex alumina injector for the analysis of acetic acid, nitrate, nitrite, sulfate, and bromide ion. The interval of the large-volume sampling was 15–30 days.

Ethyl alcohol and BTEX compounds were tested through a gas chromatograph (agilent 6890N) with a flame ionization detector. The chromatographic conditions were as follows: column temperature, 50°C (5 min), at rates of 20 and 150°C/min; injection port-temperature 200°C; detector temperature, 250°C; flow rate of carrier nitrogen, 1.0 mL/min; headspace bottle temperature, 60°C; equilibrium time, 10 min; frisking time, 5 min; clamping time, 0.13 min; injecting time, 1 min; lower limit of ethyl alcohol detection, 0.1 mg/L; and limit of BTEX detection, 0.002 mg/L.

Acetic acid, nitrate, nitrite, sulfate, and bromide ion were measured through an ion chromatograph (Dionex ICS-1000); the detection limit was 0.1 mg/L. The chromatographic conditions were column temperature, 30°C; tank temperature, 35°C; suppressor type-ASRS-4MM, SRS current, 50 mA; leachate flow rate (3.5 mmol Na_2_CO_3_ and 1 mmol NaHCO_3_), 1.20 mL/min; and sample size, 25 *μ*L.

Dissolved oxygen, pH, and water temperature were measured in site through water-level observation holes (w1–w10) using electrodes matching the multifunctional water quality analyzer made in Italy (HANAA, HI9804D).

## 3. Results and Discussion 

### 3.1. Distribution Law of BTEX Pollution Halo

**Figure 2:**
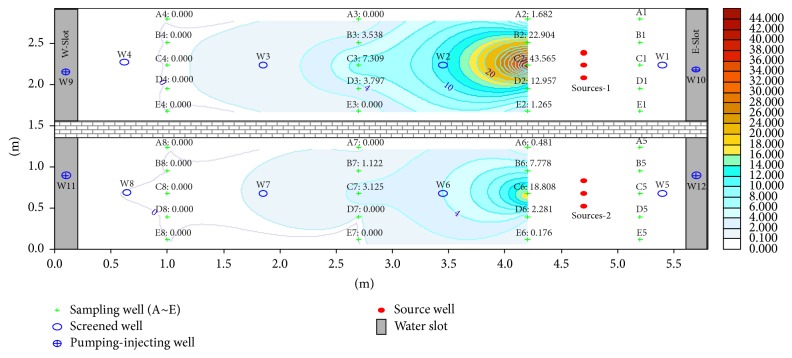
Distribution of BTEX pollution halo on the 30th day.

**Figure 3 fig3:**
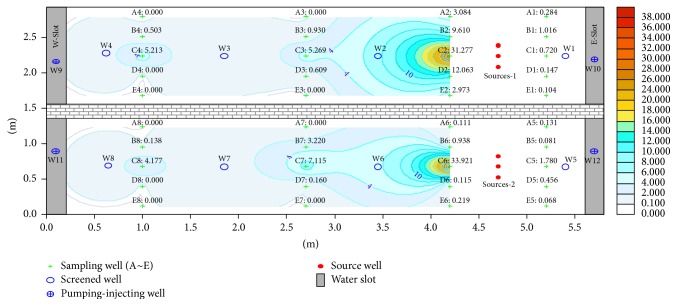
Distribution of BTEX pollution halo on the 56th day.

**Figure 4:**
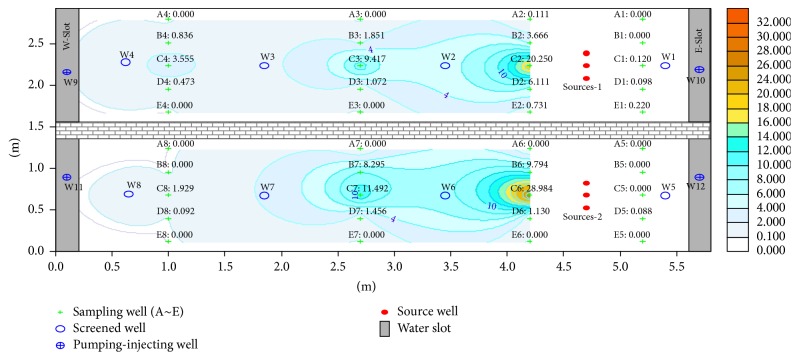
Distribution of BTEX pollution halo on the 71th day.

Fuel was filled at 45 cm depth; the BTEX content in the tank at this height corresponded to the highest value and dropped at 30 and 15 cm from the monitoring results. The distribution of the BTEX pollution halo in the tank during the first 71 days is shown in Figures 2, 3, and 4.

Figures 2 [Fig fig3]–4 reveal that the BTEX contents at C2-45 in the north tank on the 30th, 56th, and 71th days are 43.565, 31.277, and 20.250 mg/L, respectively, and the content reaches the peak during 30–60 days and declined afterwards. In the south tank where ethanol gasoline was filled, the contents at C6-45 on the 30th, 56th, and 71th days were 18.808, 33.921, and 28.894 mg/L, respectively. Because of ethyl alcohol [14], the pollution halo is small in this tank and the content peak lags behind. However, with time progression, the pollution halo shrank inward because of volatilization [15–17], adsorption [18, 19], and biodegradation [20–22]. As a result, BTEX content diminished.

**Figure 5:**
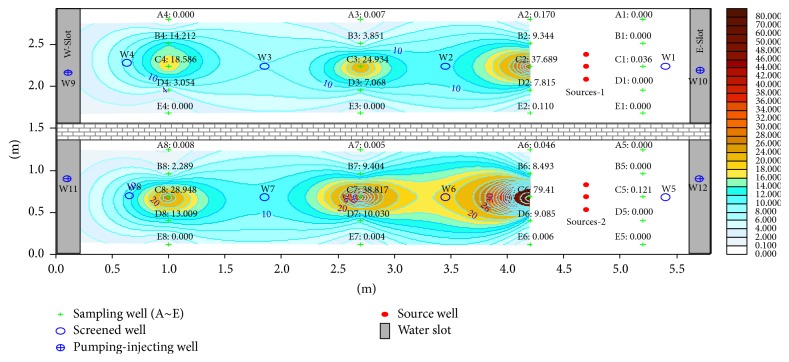
Distribution of BTEX pollution halo on the 118th day.

In the first 78 days, the underground water level remained at 45–50 cm in the sand tank. To simulate the influences of rain on BTEX, we added water in the tank until the 69 cm level was reached.  Figure 5 shows the distribution of the BTEX pollution halo on the 118th day, from which the BTEX content clearly rose, especially in the south tank, reaching as high as 79.41 mg/L. This result indicates that the rise of water level after rainfall achieved fuller contact between gasoline near the pollution source and underground water. Consequently, a great amount of BTEX dissolved in the water. The increased pollutants content deteriorated the underground water quality.

### 3.2. Longitudinal Distribution Law of BTEX

**Figure 6:**
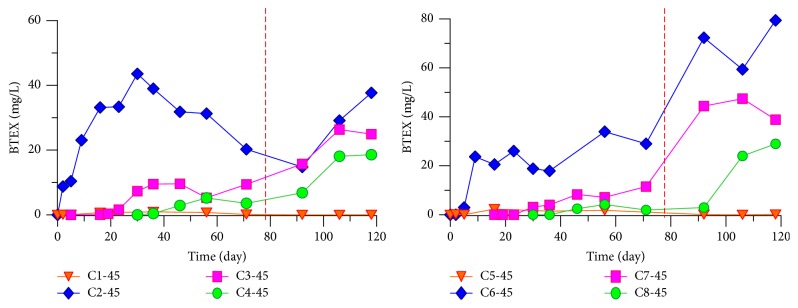
Longitudinal monitoring results of BTEX pollutant concentration on C row at the same depth.

Water sample monitoring results show that, at the same depth, the BTEX content on the C row of central axis was the highest and decreased along two sides, at the order of C > B > A and C > D > E. The longitudinal monitoring results of the BTEX pollutant concentration on the C row at 45 cm depth are shown in Figure 6.

Figure 6 reveals that BTEX was detected but with the lowest content upstream of pollution sources C1 and C5, where the pollutants mainly originated from the dispersion. By contrast, particularly downstream, the contents at the same height dropped (C2 > C3 > C4 and C6 > C7 > C8) because of the underground water flow.

### 3.3. Vertical Distribution Law of BTEX

**Figure 7:**
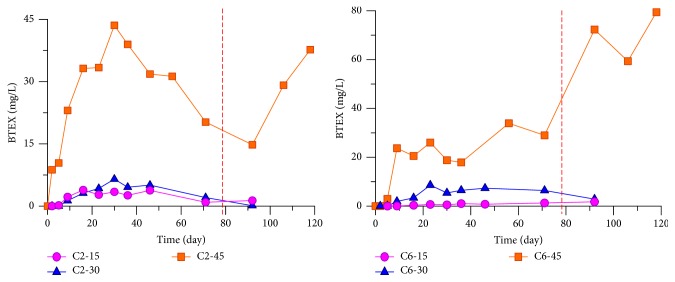
Vertical monitoring results of BTEX pollutant concentration on C2 and C6 in the sand tank.

The vertical migration and distribution of BTEX on each row were almost identical between the north (traditional gasoline) and the south (ethanol gasoline) tanks. At the C row, the content was highest but declined along two sides. For instance, in C2 of the north tank and C6 of the south tank, the vertical monitoring results of the BTEX pollutant concentration at the depths 15, 30, and 45 cm are shown in Figure 7.

Figure 7 shows that, in either tank, the BTEX content was the highest at the 45 cm depth, followed by the 30 and 15 cm depths. 78 days after adding water, the content at 45 cm depth rose more rapidly than those at the 30 and 15 cm levels, which only changed slightly. The density of BTEX is lower than that of water; hence, downward vertical migration was rarely observed, and BTEX contents were found at lower water levels.

### 3.4. Declining Features of BTEX by Biodegradation

Biodegradation is a major mechanism of destructive declination which can essentially remove pollutants by transforming pollutants in aquifers from macromolecules into small molecules or converting toxic compounds into nontoxic substances. Degradation is classified based on electron acceptor type; the respiratory action of a microorganism is called “aerobic degradation” when oxygen is used as the electron acceptor; “anaerobic degradation” for nitrate, ferric ion, sulfate, or carbon dioxide; and “facultative degradation” for dissolved oxygen and nitrate.

Microbial activity is active and common in an underground environment, including those of simple microorganisms, such as prokaryotic bacteria and cyanobacteria, as well as complicated eukaryotic algae, fungi, and prokaryotes. Since the 1970s, 28 kinds of bacteria and fungi capable of degrading hydrocarbon were extracted from the underground environment [23]. In aerobic conditions, almost all of the oil-hydrocarbon can be degraded by organisms [24]. In the underground water which is seriously short of dissolved oxygen, microorganisms can degrade petroleum hydrocarbon through electron acceptors, such as nitrate, ferric irons, and sulfate, resulting in reduction reactions.

#### 3.4.1. Changes of Dissolved Oxygen

The dissolved oxygen in each sample was detected. In the first 92 days, the injector (early stage) and peristaltic pump (middle stage) were used in sampling. Hence, aeration occurred in the water sample. As a result, the detected values were higher than the actual levels and did not reflect the real situation. After 92 days, dissolved oxygen was detected through a probe from the water-level monitoring hole. The results are shown in Table 1.

The background value of dissolved oxygen at each hole was 3–5 mg/L. After gasoline was poured, the content increased upstream the pollution source (water continued flowing in), but the dissolved oxygen was almost consumed downstream. This observation indicates that biodegradation with oxygen took place in the tank and some BTEX was degraded [25–27].

#### 3.4.2. Changes of NO_3_^−^, SO_4_^2−^, and NO_2_^−^ Contents

**Figure 8:**
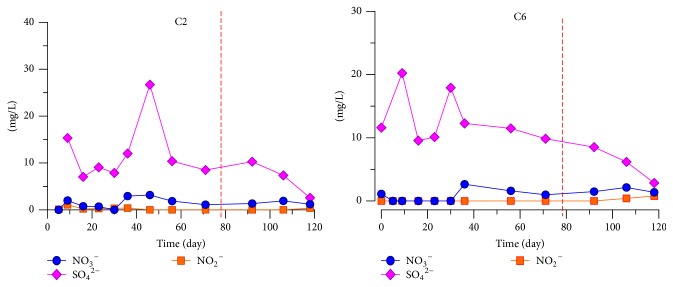
Monitoring results of electron acceptors on C2 and C6 at the 45 cm depth in the sand tank.

The fuel was filled to the 45 cm level; hence, the BTEX content at this point was the highest, whereas the contents of NO_3_^−^ and SO_4_^2−^ were basically the same. The concentration monitoring results from C2-45 and C6-45 are displayed in Figure 8.

Figure 8 shows that the contents of NO_3_^−^ and NO_2_^−^ at C2-45 and C6-45 are much lower, approximately 0, implying that biodegradation with nitrate occurred in the tank when NO_3_^−^ was degraded as the electron acceptor. When the dissolved oxygen was nearly consumed, some anaerobes can replace O_2_ with NO_3_^−^ to serve as the final electron acceptor for later hydrocarbon degradation [25–27]. The content of SO_4_^2−^ was elevated initially but fluctuated slightly after filling with water close to the background value. The content then began to drop on the 91st day to 0 on the 118th day. This observation implies that oxidation-reduction environment changed from oxidation to a reduction environment, when biodegradation with sulfate ensured. When some electron acceptor with strong oxidizability is consumed, SO_4_^2^ serves as the electron acceptor in the hydrocarbon degradation of contaminants by anaerobes. For these reasons, BTEX is degraded [25–27].

## 4. Conclusions

The BTEX content near the injection point was the highest. The lateral dispersion of the BTEX pollution halo laterally extended continuously. Longitudinally, the BTEX pollution migrated downstream mainly because of the water flow and longitudinal dispersion. Given the slight vertical dispersion, BTEX content reached the highest level at the 45 cm depth but dropped successively at the 30 and 15 cm depths. Meanwhile, effect of ethyl alcohol caused the concentration peak of BTEX in ethanol gasoline in the underground water to lag behind that of tradition gasoline.

When the underground water level rose, the BTEX content at 45 cm increased more rapidly than those at 30 and 15 cm, which changed only slightly. The density of BTEX is lower than that of water; hence, vertical migration downward was rarely observed, and lower contents were found at lower water levels.

The geochemical characteristics of the underground water environment revealed that downstream the pollution source, the concentration of dissolved oxygen and NO_3_^−^ decreased more rapidly, whereas the concentration of SO_4_^2−^ changed slightly initially and then decreased slowly. This observation indicated that oxidation, denitrification, and desulfurization took place. The fall of the BTEX content in the underground water was related to many kinds of biodegradations, such as biodegradation with oxygen, nitrate, and sulfate. Thus, alleviating BTEX pollution in underground water by increasing the concentrations of electric acceptors, such as nitrate, and by enhancing microbial activities is an effective and noteworthy method.

## Figures and Tables

**Table 1:** The monitoring results of dissolved oxygen concentration in the water level monitoring hole of sand tank (mg/L).

Time (day)	W1	W2	W3	W4	W5	W6	W7	W8
92	6.43	0.23	0.2	0.17	7.19	0.33	0.06	0.13
106	6.98	0.23	0.24	0.72	6.73	1.07	0.91	1.06
117	7.61	1.11	0.47	0.5	7.92	1.43	0.78	0.32
142	8.0	1.0	0.5	0.3	8.1	0.8	0.4	0.3
